# Significance of Thoracic Vertebral Bone Marrow CT Value Detection in the Diagnosis of Senile Osteoporosis and Assessment of Companion Thoracic Vertebral Fracture Risk

**DOI:** 10.7759/cureus.92611

**Published:** 2025-09-18

**Authors:** Laichong Huang, Weikuan Xue, Fuhui Huang, Fubin Zhang, Bin Pan, Jinchun Chen

**Affiliations:** 1 Radiology, Rui'an People's Hospital, Ruian, CHN

**Keywords:** bone marrow, ct value detection, senile osteoporosis, vertebral fracture, vertebral trabecular bone

## Abstract

Objective: This study investigates the diagnostic significance of CT values of thoracic vertebral trabecular bone in diagnosing senile osteoporosis and assessing the risk of concomitant thoracic vertebral fractures.

Methods: A retrospective analysis was conducted on 124 elderly patients diagnosed with osteoporosis (osteoporosis group), 30 patients with osteopenia (osteopenia group), and 30 individuals with normal bone mineral density (normal group) at our hospital. All participants underwent low-dose chest CT and dual-energy bone densitometry. The osteoporosis group was further divided into a fracture subgroup (42 cases) and a non-fracture subgroup (82 cases) based on the presence of thoracic vertebral fractures.

Results: CT values of thoracic vertebral trabecular bone showed a positive correlation with bone mineral density T-scores (P < 0.05). Significant differences were observed in CT values and T-scores among the three groups (P < 0.05), with the following order: osteoporosis group < osteopenia group < normal group (P < 0.05). The fracture and non-fracture subgroups also exhibited significant differences in CT values and T-scores (P < 0.05). The 95% confidence interval upper limits for CT values in the fracture subgroup, non-fracture subgroup, and osteopenia group were 54.0 HU, 85.3 HU, and 112.2 HU, respectively.

Conclusion: CT values of thoracic vertebral trabecular bone are closely associated with senile osteoporosis and hold significant diagnostic value for both osteoporosis and fracture risk assessment. A CT value < 85.3 HU may serve as a diagnostic threshold for osteoporosis, while a CT value < 54.0 HU indicates an increased risk of thoracic vertebral fractures.

## Introduction

The CT values of trabecular bone in lumbar vertebrae are correlated with bone density T-scores, while there is no correlation with the CT values of cortical bone [1]. Using the CT values of trabecular bone in lumbar vertebrae to diagnose osteoporosis shows good accuracy and sensitivity, providing a certain reference value [2]. However, there is a lack of relevant literature on the correlation between the CT values of trabecular bone in thoracic vertebrae and bone density T-scores, the application of CT values of trabecular bone in thoracic vertebrae in the diagnosis of osteoporosis, and the value of CT values of trabecular bone in thoracic vertebrae in assessing the risk of osteoporosis accompanied by vertebral fractures. This study retrospectively collected data from 124 elderly patients diagnosed with senile osteoporosis and 35 patients with normal bone density (normal group), as well as 30 patients with reduced bone mass (reduced bone mass group) who underwent low-dose chest CT and dual-energy bone densitometry at our hospital. The osteoporosis group was divided into a fracture group (42 cases) and a non-fracture group (82 cases) based on the presence of vertebral fractures. This study aims to explore the correlation between the CT values of trabecular bone in thoracic vertebrae and bone density T-scores, as well as the value of CT values of trabecular bone in thoracic vertebrae in the diagnosis of senile osteoporosis and in assessing the risk of vertebral fractures.

## Materials and methods

General information

This retrospective study included 124 elderly patients diagnosed with senile osteoporosis (osteoporosis group) and 35 patients with normal bone density (normal group) who underwent low-dose chest CT and dual-energy bone densitometry at our hospital, as well as 30 patients with reduced bone mass (reduced bone mass group). The senile osteoporosis group was divided into a fracture group (42 cases) and a non-fracture group (82 cases) based on the presence of vertebral fractures. In the fracture group, there were 22 males and 20 females, aged 70 to 86 years, with an average age of 76.8 ± 4.7 years; a total of 78 thoracic vertebrae had fractures. In the non-fracture group, there were 42 males and 40 females, aged 70 to 87 years, with an average age of 77.2 ± 4.8 years. In the reduced bone mass group, there were 18 males and 17 females, aged 71 to 86 years, with an average age of 76.9 ± 4.7 years. In the normal group, there were 16 males and 14 females, aged 70 to 85 years, with an average age of 76.1 ± 4.6 years. There were no statistically significant differences in gender and age among the four groups (all P > 0.05). Exclusion criteria included individuals with metabolic bone diseases, chronic kidney disease, infectious bone diseases, multiple myeloma, osteogenesis imperfecta, and benign or malignant bone tumors. This study was approved by the hospital’s ethics committee, and all participants signed informed consent forms.

Methods

Bone Density T-score Measurement

The bone mineral density (BMD) of the thoracic vertebrae was measured using the LUNAR PRODIGY dual-energy X-ray bone density scanner (GE HealthCare, Madison, Wisconsin). The diagnostic criteria for osteoporosis were defined as follows: T-score ≤ -2.5 for osteoporosis; T-score between -1.0 and -2.5 for osteopenia; and T-score ≥ -1.0 for normal bone density.

Chest CT Examination

The chest CT scans were performed using the United Imaging UCT-510 64-slice CT machine, with a full lung breath-hold scan covering thoracic vertebrae T1 to T12. The parameters were set as follows: voltage 100 kV; tube current 30 mA (automatically adjusted by DOM); rotation time 0.5 s; collimator width 40 mm; pitch 1.0875; slice thickness 5 mm; slice interval 5 mm; matrix size 512 × 512; field of view (FOV) 280-330 mm. A bone algorithm (B_SHARP_C) was employed for reconstruction with a slice thickness of 1.0 mm and a corresponding slice interval. The window width was set to 1800, and the window level to 600.

Thoracic Vertebra CT Value Measurement

The Green Lander-PACS software (Zhejiang Greenlander Information Technology Co. Ltd., Hangzhou, China) provided tools for measuring the cancellous bone CT values of thoracic vertebrae T4, T6, and T12 (if fractures were present, measurements were taken 1-2 vertebrae above or below). CT values were measured within a region of interest with a diameter of 5 mm, and the measurement tool was copied and pasted to assess the next vertebra’s CT value. The average of CT values from the three measured vertebrae was computed.

Observation Indicators

The correlation between the CT values of thoracic vertebrae and bone density T-scores was analyzed, differences in CT values of trabecular bone in thoracic vertebrae and bone density T-scores among the groups were compared, and the 95% confidence interval for the CT values of trabecular bone in thoracic vertebrae in the diagnosis of osteoporosis and in cases accompanied by vertebral fractures was explored.

Statistical Methods

Statistical analysis was performed using IBM SPSS Statistics for Windows, Version 19 (Released 2010; IBM Corp., Armonk, New York). Data were expressed as mean ± standard deviation and analyzed using paired-samples t-test and one-way ANOVA. The correlation between two samples was analyzed using bivariate correlation. P < 0.05 indicates that the difference is statistically significant.

## Results

Correlation between CT values of trabecular bone in thoracic vertebrae and bone density T-scores 

**Figure 1:**
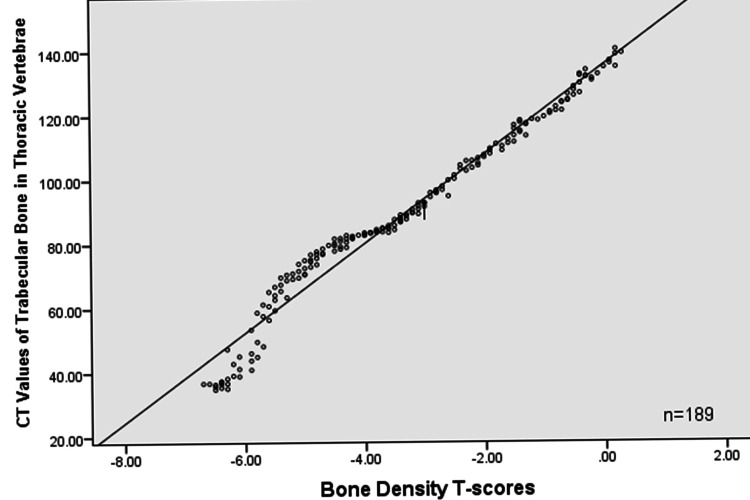
Correlation between CT values of trabecular bone in thoracic vertebrae and bone density T-scores

In 189 cases, the CT values of trabecular bone in thoracic vertebrae showed a positive correlation with bone density T-scores (r = 0.984, all P < 0.05), as shown in Figure 1.

Comparison of CT values of vertebral trabecular bone and bone density T-scores among osteoporosis, low bone mass, and normal groups

The CT values of vertebral trabecular bone and bone density T-scores for the osteoporosis group, low bone mass group, and normal group were 72.3 ± 18.5, 110.3 ± 5.7, 129.5 ± 5.9 and -4.6 ± 1.2, -1.8 ± 0.4, -0.4 ± 0.4, respectively. The differences in all observed parameters among the three groups were statistically significant (F = 288.183 and 403.225, both P < 0.05). The CT values of vertebral trabecular bone and bone density T-scores followed the trend: osteoporosis group < low bone mass group < normal group, with all pairwise comparisons showing statistically significant differences (all P < 0.05) (see Table 1).

**Table 1:** Comparison of CT values of vertebral trabecular bone and bone density T-scores among osteoporosis, low bone mass, and normal groups ^a^Compared with the low bone mass group and normal group, t = 64.543, 93.854 and 50.125, 103.585, all P＜0.05. ^b^Compared with the normal group, t = 54.986 and 49.000, both P＜0.05.

Category	n	CT Value of Vertebral Trabecular Bone	Bone Density T-score
Osteoporosis group	124	72.3±18.5^a^	-4.6 ±1.2^a^
Low bone mass group	35	110.3 ±5.7^b^	-1.8±0.4^b^
Normal group	30	129.5±5.9	-0.4±0.4
F		288.183	403.225
P		0.000	0.000

Comparison of CT values of vertebral body trabecular bone and bone mineral density T-scores between the fracture group and the non-fracture group in elderly patients with osteoporosis

The CT values and bone mineral density T-scores for the fracture group and non-fracture group were 51.1 ± 12.5, 83.7 ± 7.3 and -5.9 ± 0.4, -3.9 ± 0.8, respectively. The differences in all observed indicators between the two groups were statistically significant (t = 31.775 and 108.648, both P < 0.05) (see Table 2).

**Table 2:** Comparison of CT values of trabecular bone in thoracic vertebrae and bone density T-scores between the fracture group and the non-fracture group in elderly patients with osteoporosis

Category	n	CT Values of Thoracic Vertebrae（HU）	Bone Density T-scores
Fracture group	42	51.1±12.5	-5.9±0.4
Non-fracture group	82	83.7±7.3	-3.9±0.8
t		31.775	108.648
P		0.000	0.000

Analysis of 95% confidence intervals for CT values of vertebral trabecular bone in the fracture group, non-fracture group, low bone mass group, and normal group

The upper limits of the 95% confidence intervals for CT values of vertebral trabecular bone in the fracture group, non-fracture group, and low bone mass group were 54.0 HU, 85.3 HU, and 112.2 HU, respectively.

## Discussion

Correlation between CT values of trabecular bone in thoracic vertebrae and bone density T-scores in elderly osteoporotic patients

Globally, there are approximately 8.9 million cases of osteoporotic fractures each year, averaging one fracture every three seconds [3]. With the continuous aging of the world population, the incidence of senile osteoporosis is gradually increasing, becoming a significant global health issue [4]. Osteoporosis is typically only evident on standard X-ray films when bone density loss exceeds 30%. The diagnosis of osteoporosis using standard X-ray films lacks objective diagnostic criteria and relies solely on clinical experience, which is unreliable and cannot achieve early diagnosis. Dual-energy X-ray absorptiometry (DEXA) is the gold standard for diagnosing osteoporosis. In our city, with a population of 1.4 million, there is only one dual-energy bone densitometer and 28 CT machines. This indicates that the availability of dual-energy bone densitometers in primary hospitals is lower than that of CT machines. Using CT machines to replace dual-energy bone densitometers for diagnosing osteoporosis could rapidly enhance the diagnosis rate and accessibility of osteoporosis assessments.

CT values refer to the density values of tissues presented during CT scans, typically measured in Hounsfield Units (HU). The bone density T-score is obtained through DEXA and reflects an individual’s bone density compared to a young healthy population; a lower T-score indicates a higher risk of osteoporosis. CT values can serve as a surrogate indicator for bone density T-scores, aiding in improving the diagnostic efficiency of osteoporosis [5]. The correlation between CT values and bone density T-scores provides new insights for the early diagnosis of osteoporosis [6]. CT values can be an effective tool for assessing bone density [7]. Changes in CT values are closely related to changes in bone density T-scores, indicating their significance in the assessment of osteoporosis [8]. The application of CT technology provides important imaging evidence for the early diagnosis of osteoporosis [9]. The correlation between CT values and bone density exists across different age groups [10]. The trends in changes of CT values and bone density are similar, highlighting their importance in clinical evaluation [11].

This study suggests a positive correlation between the CT values of trabecular bone in thoracic vertebrae and bone density T-scores (P < 0.05). There may be differences in CT values between the upper and lower thoracic vertebrae, which require further confirmation with larger sample sizes. We assessed the average CT values of trabecular bone in the upper, middle, and lower thoracic vertebrae. The results of this study indicate that measuring the CT values of trabecular bone in thoracic vertebrae can serve as an assessment method for osteoporosis. However, there is a lack of relevant literature regarding the threshold for diagnosing osteoporosis using CT values of trabecular bone in thoracic vertebrae and their value in assessing the risk of vertebral fractures in patients with osteoporosis.

The value of thoracic vertebral bone marrow CT value detection in the diagnosis of senile osteoporosis

CT is a high-resolution imaging examination method that can clearly display the microstructure of bones. The CT value is an objective indicator reflecting changes in tissue density in the human body; a high CT value indicates high tissue density, while a low CT value indicates low tissue density. Measuring the CT value of trabecular bone can objectively express the quality of trabecular bone. The variation in CT values is similar to the trend in changes in bone density, highlighting its importance in clinical evaluation [12]. The application of CT technology provides significant imaging evidence for the early diagnosis of osteoporosis [13-16]. Previous studies rarely mentioned comparing the CT values of vertebral trabecular bone as independent observational objects with bone density T-scores.

Bone can be categorized into trabecular and cortical bone. In patients with osteoporosis, the difference in structure between cortical and trabecular bone may lead to different rates of bone loss during the process of bone quality deterioration. Therefore, we compared the CT values of vertebral trabecular bone as independent variables with bone density T-scores. The results showed that there were statistically significant differences in the CT values of vertebral trabecular bone and bone density T-scores among the osteoporosis group, low bone mass group, and normal group (all P < 0.05). Furthermore, both the CT values of vertebral trabecular bone and bone density T-scores followed the trend: osteoporosis group < low bone mass group < normal group, with statistically significant differences in pairwise comparisons (all P < 0.05). This indicates that measuring the CT values of vertebral trabecular bone can serve as an objective criterion for diagnosing senile osteoporosis, although the diagnostic range values require further investigation.

The value of thoracic vertebral bone marrow CT value detection in the diagnosis of senile osteoporosis and assessment of thoracic vertebral fracture risk

The correlation between CT values and bone density indicates that CT values can effectively reflect changes in bone density, providing a basis for the early diagnosis of osteoporosis [17-20]. CT has high sensitivity and specificity in assessing spinal fractures in patients with osteoporosis, offering effective diagnostic evidence for clinical practice [18-24]. The importance of CT measurements in the diagnosis of osteoporosis and the assessment of fracture risk suggests that CT values can serve as effective predictive indicators for osteoporosis [25]. CT can effectively evaluate vertebral bone quality in elderly patients with osteoporosis, highlighting the significance of CT in clinical applications [26]. The clinical significance of CT imaging in patients with osteoporosis indicates that the correlation between CT values and bone density provides important references for clinical practice [27-30]. There is no relevant literature reported on the assessment of the risk of thoracic vertebral fractures in elderly patients with osteoporosis by measuring the CT values of the thoracic vertebrae.

The study found statistically significant differences (all P < 0.05) in the CT values of the thoracic vertebrae and T-scores of bone density between the fracture group and the non-fracture group. Therefore, measuring the CT values of the thoracic vertebrae can serve as a risk assessment indicator for thoracic vertebral fractures in elderly patients with osteoporosis. This provides an objective evaluation index for the assessment of fractures in elderly patients with osteoporosis. Early intervention for elderly patients with osteoporosis has significant clinical value in improving their quality of life.

This study analyzed the 95% confidence intervals of thoracic vertebral bone marrow CT values in the fracture group, non-fracture group, and bone mass reduction group. The results suggested that a thoracic vertebral bone marrow CT value of less than 112.2 HU could be used as a diagnostic criterion for bone mass reduction. Additionally, a thoracic vertebral bone marrow CT value of less than 85.3 HU could serve as a diagnostic standard for senile osteoporosis. Furthermore, if the thoracic vertebral bone marrow CT value is less than 54.0 HU, the risk of thoracic vertebral fractures would increase. These findings are for reference only and require further confirmation through larger sample data.

Significance and limitations

Assessment of osteoporosis using thoracic vertebral CT values can help avoid repeated bone density scans for patients who have already undergone chest CT examinations, thereby reducing unnecessary X-ray radiation exposure and lowering medical costs, which is of significant clinical value. Using thoracic vertebral CT values to evaluate fracture risk can alert elderly osteoporosis patients to initiate preventive measures and treatment in advance, thereby reducing fracture risk.

However, this study has limitations: the sample size was relatively small, and further validation with larger datasets is needed. Additionally, the study did not compare differences in thoracic vertebral CT values between genders or among different vertebral segments in osteoporosis patients, which warrants further investigation.

## Conclusions

There is a positive correlation between CT values of thoracic vertebral trabecular bone and T-scores of bone mineral density. The measurement of thoracic vertebral trabecular bone CT values holds a significant reference value for diagnosing senile osteoporosis and assessing the risk of thoracic vertebral fractures.
